# Biological Control Products for Aflatoxin Prevention in Italy: Commercial Field Evaluation of Atoxigenic *Aspergillus* *flavus* Active Ingredients

**DOI:** 10.3390/toxins10010030

**Published:** 2018-01-05

**Authors:** Antonio Mauro, Esther Garcia-Cela, Amedeo Pietri, Peter J. Cotty, Paola Battilani

**Affiliations:** 1 International Institute of Tropical Agriculture, P.O. Box 34441 Dar es Salaam, Tanzania; a.mauro@cgiar.org; 2 Applied Mycology Group, Environment and AgriFood Theme, Cranfield University, Cranfield, Bedford MK43 0AL, UK; m.e.garcia-cela@cranfield.ac.uk; 3 Institute of Food Science and Nutrition, Università Cattolica del Sacro Cuore, 29100 Piacenza, Italy; amedeo.pietri@unicatt.it; 4 United States Department of Agriculture, Agricultural Research Service, School of Plant Sciences, University of Arizona, Tucson, AZ 85721, USA; Peter.Cotty@ARS.USDA.GOV; 5 Department Sustainable Crop Production, Università Cattolica del Sacro Cuore, 29100 Piacenza, Italy

**Keywords:** biopesticide, biocontrol, mycotoxin, fumonisin, maize, VCG, SSR, AF-X1™, aflatoxin prevention, Europe, The first active ingredient for biological control of aflatoxins in maize produced in Europe was selected. Commercial field trials in Italy support further development and registration of the biopesticide named AF-X1^TM^.

## Abstract

Since 2003, non-compliant aflatoxin concentrations have been detected in maize produced in Italy. The most successful worldwide experiments in aflatoxin prevention resulted from distribution of atoxigenic strains of *Aspergillus* *flavus* to displace aflatoxin-producers during crop development. The displacement results in lower aflatoxin concentrations in harvested grain. The current study evaluated in field performances of two atoxigenic strains of *A*. *flavus* endemic to Italy in artificially inoculated maize ears and in naturally contaminated maize. Co-inoculation of atoxigenic strains with aflatoxin producers resulted in highly significant reductions in aflatoxin concentrations (>90%) in both years only with atoxigenic strain A2085. The average percent reduction in aflatoxin B_1_ concentration in naturally contaminated maize fields was 92.3%, without significant differences in fumonisins between treated and control maize. The vegetative compatibility group of A2085 was the most frequently recovered *A. flavus* in both treated and control plots (average 61.9% and 53.5% of the *A. flavus*, respectively). A2085 was therefore selected as an active ingredient for biocontrol products and deposited under provisions of the Budapest Treaty in the Belgian Co-Ordinated Collections of Micro-Organisms (BCCM/MUCL) collection (accession MUCL54911). Further work on development of A2085 as a tool for preventing aflatoxin contamination in maize produced in Italy is ongoing with the commercial product named AF-X1™.

## 1. Introduction

Aflatoxins, in particular aflatoxin B_1_, are among the most toxic natural compounds with demonstrated carcinogenic effect on humans [1]. Aflatoxins are secondary metabolites produced by several species of *Aspergillus* on important commodities including maize, cottonseed, peanuts, and pistachio nuts [2,3]. *Aspergillus flavus* is the fungus most frequently implicated as the causal agent of aflatoxin contamination in maize worldwide, and the primary etiologic agent on maize in Italy [4]. Maize production in Italy is mainly located in five districts (90% of national production; [5]) placed in the north of the peninsula where weather conditions are commonly unfavorable for aflatoxin contamination. However, poor rainfall and increased temperature, as occurred during the 2003 maize season, may lead to aflatoxin contamination above legal limits for humans and dairy animals [6]. Indeed, in 2003 non-compliant aflatoxin concentrations were detected in cow’s milk [4,7].

Although several strategies have been applied worldwide to reduce pre-harvest aflatoxins contamination, biological control with atoxigenic strains of *A*. *flavus* distributed in field to reduce pre-harvest aflatoxin contamination is both highly effective and readily adapted by industry [8,9,10,11]. Atoxigenic strains displace aflatoxin producers during crop development resulting in significant reductions in aflatoxin contamination in the harvested grain [12,13,14,15]. The efficacy of atoxigenic *A*. *flavus* as biocontrol agents is well documented in several crops worldwide [10,12,16,17,18,19,20,21]. Cotty [22] reported for the first time the use of an atoxigenic strain to reduce aflatoxin B_1_ concentrations in a developing crop. This was followed by field trials in which atoxigenic strain applications caused both increased incidences of the applied atoxigenic strain on the crop and associated reductions in aflatoxin concentrations [23]. Both Brown et al. [24] and Abbas et al. [25] observed reductions in aflatoxin B_1_ contamination between 65% and 95% in corn fields. Similar results were obtained in Italy in an experimental field trial where reductions greater than 80% were achieved using bioplastic granules carrying an atoxigenic strain of *A*. *flavus* selected in USA [26].

*Aspergillus flavus* has a vegetative incompatibility system [27], probably evolved to limit hyphal fusion, virus transmission and gene flow between individuals belonging to different vegetative compatibility groups (VCGs) [28]. Vegetative compatibility analyses are a powerful instrument to characterize *A*. *flavus* populations [29,30,31], to describe the diversity or to track the fate of a specific VCG, as a VCG applied as biocontrol agent in field [10,23]. Since 2003, several studies have been conducted to understand and characterize the *A*. *flavus* population in maize in Italy and to predict the behavior in field [4,7,32,33,34] with the main aim to select candidate biocontrol agents. Based on encouraging results obtained, the current study focuses on the evaluation of in field performances of two selected atoxigenic strains in artificially inoculated maize ears in 2012 and 2013 and in maize fields naturally contaminated in 2012.

## 2. Results

### 2.1. Pin-Bar Inoculation Experiment

Aflatoxin contamination of unwounded and uninoculated controls was similar in the two years with 0.7 and 0.6 μg aflatoxin B_1_/kg in 2012 and 2013, respectively. Aflatoxin B_1_ was dominant among the detected aflatoxins; therefore, this is the only compound reported. Ears inoculated aflatoxin producer *A*. *flavus* strain A2092 produced significantly (*p* = 0.05) less aflatoxin B_1_ in 2013 (133 μg/kg) than in 2012 (1415 μg/kg). In ears inoculated with an atoxigenic isolate alone (either *A*. *flavus* strain A2085 or *A*. *flavus* strain A2321), aflatoxin B_1_ concentration did not differ significantly from that of the uninoculated controls. Co-inoculation of wounded maize ears with aflatoxin-producer A2092 and atoxigenic isolate A2321 did not result in aflatoxin concentrations significantly different from inoculation with A2092 alone as reported in Table 1. On the other hand, co-inoculation of atoxigenic strain A2085 with A2092 resulted in highly significant reductions in aflatoxin concentrations of greater than 90% in both years.

### 2.2. Field Application of Atoxigenic Aspergillus flavus Strains

#### 2.2.1. *Aspergillus flavus* Population

The *A*. *flavus* population ranged between 4.82 ln colony forming unit (CFU)/g and 6.82 ln CFU/g in control plots and from 5.52 ln CFU/g and 7.18 ln CFU/g in the treated plots as reported in Table 2. On average, the population appeared higher in the plots treated with the two Italian atoxigenic strains compared to the untreated plots (6.51 ln CFU/g vs. 6.16 ln CFU/g). Differences were observed between fields; in particular, field VR1 had the highest fungal population (7.00 ln CFU/g) and fields MN2 and RO3 the lowest (5.39 ln CFU/g and 5.31 ln CFU/g, respectively).

Vegetative compatibility analyses were conducted on 60 isolates from each field: 30 from untreated and 30 from the treated areas. The percentage of the VCG IT019 in the untreated plots ranged from 16.7% to 35.0% (average 25.2%) and the VCG IT006 from 35.0% to 73.7% (average 53.5%). In the plots treated with the atoxigenic strains, the percentage of recovery of VCG IT019 was 16.7–46.7% (average 30.0%) and for the VCG IT006 the range was 40.0–76.7.0% (average 61.9%). Members of VCG IT019 and IT006 were recovered in higher percentage in five and six of the eight fields treated, respectively, compared to the control; in three fields (MN2, PR, VR2) the frequency of both VCGs were higher in treated plots than in untreated controls. In almost all fields the application of the candidate biocontrol agents reduced the presence of *A*. *flavus*, not in one of the two applied atoxigenic VCGs compared to the untreated control, as reported in Table 2. A significant negative correlation between IT006 and IT019 was found in treated plots (r = −0.91; *p* ≤ 0.001), no correlation was detected in untreated plots.

#### 2.2.2. Mycotoxin Contamination

In four of the test locations (Fields RO1, MN1, MN2, & RO3) aflatoxin B_1_ concentrations were less than 1.0 μg/kg in both treated and control plots as showed in Table 3; these locations were excluded from the statistical analyses. In the other fields, average aflatoxin B_1_ concentrations ranged from 8.6 μg/kg to 150.7 μg/kg in the control, and from 0.2 μg/kg to 8.4 μg/kg in the treated area as reported in Table 3. The overall average aflatoxin B_1_ concentration in maize from the treated plots (4.2 μg/kg) was significantly less than concentrations in maize from untreated plots (71.1 μg/kg). The interaction between treatments and fields was not significant. The percent reduction in aflatoxin B_1_ concentration ranged from 83.7% to 94.8%, with an average of 92.3% as showed in Table 3. The lowest percentage of reduction was achieved in the field (PR) with the lowest concentrations of aflatoxins (mean = 8.6 μg/kg). However, no differences were observed among fields in percent aflatoxin B_1_ reduction as reported in Table 3.

Fumonisins (B_1_ + B_2_) contamination ranged from 0.1 mg/kg to 7.9 mg/kg and from 0.1 mg/kg to 11.1 mg/kg in the control and treated plots, respectively (Table 3). Observed differences in fumonisin concentration between the controls and treatments were never significant, both with single field comparisons (*p* = 0.101) and when utilizing each of the eight fields as replicates (*p* = 0.629; Table 3).

### 2.3. Mating-Type and Microsatellites

Amplification of the mating type genes revealed that strain A2321 has the idiomorph *MAT1-1* and strain A2085 has the idiomorph *MAT1-2*. Single sequence repeat (SSR) analysis at 16 microsatellite loci indicated that the two Italian atoxigenic strains are distinct both from each other and from *A*. *flavus* NRRL18543 and *A*. *flavus* NRRL21882 (the active ingredients of the biocontrol products AF36 and Aflaguard^®^, respectively). Of the 16 loci, eight (*Aspergillus Flavus* (AF) 16, AF22, AF28, AF31, AF53, AF54, AF63, and AF64) were invariable among the four *A*. *flavus* genotypes as showed in Table 4.

## 3. Discussion

Biocontrol products directed at preventing aflatoxin contamination with atoxigenic genotypes of *A*. *flavus* contain the atoxigenic active ingredient and a source of nutrients to support reproduction of the fungus. Commercial products currently consist of grain (whole wheat, whole sorghum, or pearled barley) coated with spores of the active ingredients. After application, the atoxigenic *A*. *flavus* grows on the grain (nutrient source), sporulates, and disperses to other organic matter including the target crop [35]. These solid formulations of the biocontrol fungi support both delivery to and residence in fields [36]. Sporulation on the coated grain may extend for relatively long periods and, as a result, provide windows of activity that continue considerably beyond the application date [13]. In addition, debris associated with treated crops and other colonized organic matter may extend the benefits of atoxigenics well beyond the year of treatments [8,13]. The current report documents high levels of efficacy in a biocontrol product, AFIT-01, for the prevention of aflatoxin contamination of maize that was developed based on the above principle and was formulated with atoxigenic *A*. *flavus* native to Italy as the active ingredients. The active ingredients of AFIT-01 are endemic across the Italian peninsula and, as such, this biocontrol product is adapted to and appropriate for use in the maize production regions of Italy.

In the field application studies, aflatoxin concentrations reported in control plots occurred without application of aflatoxin-producing fungi and without altering commercial production practices. Thus, in agreement with previously observed trends [37], the current study detected significant natural infection of commercial maize in Italy with detected concentrations of aflatoxin B_1_ in maize that are of concern for human food and dairy feed ranging from 8.6 μg/kg to 150.7 μg/kg. Fields producing contaminated maize were distributed across three maize producing political districts (Verona, Parma, and Rovigo) confirming prior reports [38,39] that aflatoxin contamination of maize was common in southern Europe during 2012.

Maize produced on 50% of the eight farms included in the study contained significant contamination and the contaminated fields averaged over 70 µg aflatoxin B_1_/kg. Under these natural conditions that favored unacceptable contamination in control plots, the biocontrol agents performed well reducing contamination: on average 92% (Table 3) and in all cases to below 10 µg aflatoxin B_1_/kg. The biocontrol agents were highly effective even though they were applied at an early growing stage. Such early applications enlarge the crop development window during which applications may be made and allows for the use of simple readily available equipment, like fertilizer spreaders, for product distribution. The results also demonstrate that if the farmers had utilized the biocontrol product AFIT-01 during 2012, problems with aflatoxins would have been greatly reduced. Indeed, aflatoxin contents of maize produced within plots treated with AFIT-01 in the fields with contamination had reductions in aflatoxin content ranging from 83.7% to 94.8% (Table 3). Furthermore, the proportion of the atoxigenic active ingredients in *A*. *flavus* populations was greatly increased even where no contamination was detected. Thus, even in years with little contamination, there can be positive influences on the composition of *A*. *flavus* populations from the biocontrol with advantages resulting from carry over between crops and into the next season, in addition to the movement of the atoxigenics, instead of aflatoxin producers, with the treated crop throughout the value chain [10]. The active ingredients were also recovered in the untreated area of maize crops, but aflatoxin was significantly higher than in treated areas. This could be due to the late dispersal of the atoxigenic to the maize ears, and the resulting inefficient competition during early stages of grain infection. On the other hand, the atoxigenic *A*. *flavus* genotypes could have played a role also in the untreated area, suggesting the reduction rate was underestimated.

The modified ratio of atoxigenic to aflatoxin-producing *A*. *flavus* in the fungal population resulted in reductions in aflatoxin B_1_. However, no significant changes were noticed in fumonisins, another relevant mycotoxin commonly detected in Italy [40,41]. This is expected because the mechanism of aflatoxin reduction, a change in the composition of *A*. *flavus* populations, is not supposed to influence *Fusarium verticillioides*, the causal agent of fumonisin contamination.

Pin-bar inoculation is a powerful tool for evaluating the reaction of maize to kernel infection by *A*. *flavus* [42]. In the current work, this technique was successfully applied to assess the ability of atoxigenic strains of *A*. *flavus* to reduce aflatoxin concentration produced by an aflatoxin-producer in developing maize ears. In these studies, *A*. *flavus* A2321 was not effective at reducing contamination. However, *A*. *flavus* A2085 was very effective. This suggests that A2085 is a better choice than A2321 as an active ingredient for a biological control product directed at preventing aflatoxin contamination of maize in Italy. Preference for A2085 is supported both by the wound inoculation tests in commercial maize fields in northern Italy and by the superior ability of A2085 to move up to the crop from formulated product and to displace aflatoxin-producers from the developing maize crop (Table 2). In all fields where significant contamination was detected, A2085 move up to the crop and displaced aflatoxin producers to a greater extent than A2321. Further, the ratio of IT006 to IT019 is similar across all test locations, suggesting an actual competitive difference between the two isolates in this cropping system that was not detected in laboratory tests [34].

Preference for A2085 as an active ingredient in biocontrol products directed at preventing aflatoxins in maize produced in Italy is also supported by the prior observation [32] that the VCG to which isolate A2085 belongs (VCG IT006) is also more widely distributed across maize producing regions in Italy than the VCG to which A2321 belongs (VCG IT019). The increased incidence suggests better adaption to the sampled agroecological zones [43]. Based on these results, A2085 was deposited under the provisions of the Budapest Treaty in The Belgian Co-Ordinated Collections of Micro-Organisms (BCCM/MUCL) code MUCL54911. Further work on the development of this strain as a tool for preventing aflatoxin contamination in maize produced in Italy is ongoing with the commercial product named AF-X1™.

## 4. Materials and Methods

### 4.1. Isolates of Aspergillus flavus

Atoxigenic isolates of *A*. *flavus* A2085 and A2321 were evaluated as active ingredients in the current study. Both Italian atoxigenic isolates contain a large deletion in the sub-telomere of chromosome 3 [44] that include the entire aflatoxin biosynthesis gene cluster and genes required for cyclopiazonic acid production [32,34]. *Aspergillus flavus* A2085 belongs to VCG IT006, the most prevalent VCG associated with maize produced in north Italy [32]. *Aspergillus flavus* A2321, belongs to VCG IT019, and was the most effective among 18 atoxigenic Italian *A*. *flavus* at reducing maize contamination in laboratory studies [34]. The aflatoxin-producing *A*. *flavus* A2092, isolated from maize produced in North Italy [32], was used in inoculation experiments.

For inoculum, conidia from 6-day-old cultures grown at 31 °C (dark) on 5/2 agar (5% V-8 juice, 2% agar, pH 5.2, 1000 mL H_2_O) in 9-cm Petri dishes were suspended in sterile distilled water and adjusted to 10^5^ spore/mL with a haemocytometer [23].

### 4.2. Pin-Bar Inoculation Experiment

Wound inoculation trials were performed in commercial maize fields near Piacenza (North Italy) in 2012 and 2013. In both years the medium season length hybrid Pioneer PR33M15 (FAO class 600), was inoculated using the pin-bar technique [42].

Maize was planted on 16th March and 4th May in 2012 and 2013, respectively, at rate of 7.5 plants/m^2^. Silking ears (BBCH69; stigmata completely dry; [45,46]) were wound inoculated with three needles arranged in a triangle with 2 cm on a side. Pins were dipped in a conidial suspension (10^5^ spore/mL) and pressed through the husk into developing kernels in the central portion of the ear. The treatments were: (1) not inoculated and not wounded control; (2) inoculated with A2085 alone; (3) inoculated with A2321 alone; (4) inoculated with A2092 alone; (5) co-inoculated with A2092 + A2085; (6) co-inoculated with A2092 + A2321. In 2013, a wounded, not-inoculated control was also included. For co-inoculation treatments, the aflatoxin producer was inoculated immediately prior to the atoxigenic isolate. Needles were surface sterilized in 80% ethanol (1 min) and rinsed twice in sterile distilled water between applications. Each replicate consisted of a single ear and treatments were replicated three times and arranged in a randomized complete block design.

Ears were wounded on 11th July and 6th August in 2012 and 2013, respectively.

At commercial ripening (moisture content 22–24%), inoculated ears from each replicate plot were de-husked and hand shelled in the 3-cm radius around the area of pin bar inoculation, and the kernels dried (45 °C, 3 days) and milled to produce flour.

### 4.3. Natural Contamination Experiment

#### 4.3.1. Preparation of Atoxigenic *A*. *flavus* Based Product

Test biocontrol products with the two atoxigenic isolates, A2085 and A2321, as active ingredients were produced on sterile sorghum in a manner similar to that recently described [10]. Briefly, the water content of the sorghum was increased to 25% prior to sterilization (20 min, 121 °C). The two atoxigenics were grown on the sorghum individually. Sterile sorghum grain was seeded with a spore suspension (10^7^ spores in 10 mL H_2_O), shaken by hand to evenly coat the seed, and incubated (31 °C, 2 days, dark). After incubation, the colonized grain was transferred to cotton bags and dried (45 °C, 48 h). Sorghum colonized with each atoxigenic isolate individually were blended in equal proportions to make the end use biopesticide (called AFIT-01) that was applied to fields.

#### 4.3.2. Maize Field Locations and Experimental Design

**Figure 1:**
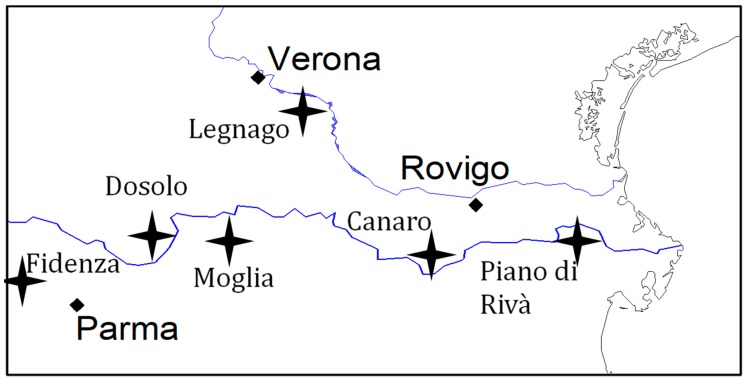
Localization of biopesticide AFIT-01 efficacy evaluation sites in North Italy.

The ability of the biopesticide AFIT-01 to prevent contamination of commercial maize during aflatoxin epidemics was evaluated in 8 locations during the 2012 maize growing season (March–September). The 8 test fields were distributed in 3 regions of north Italy (Figure 1): Emilia Romagna (ER; 1 field), Lombardia (LO; 2 fields) and Veneto (VN; 5 fields). The commercial hybrid Pioneer PR33M15 (Pioneer Hi-Bred Italia, Gadesco Pieve Delmona, Italy) used in the wound inoculation experiments above was seeded at a rate of 7.5 plants/m^2^ in all fields. Farmers managed each field, approximately 2 hectares (ha) following standard practices with 1 hectare receiving treatments and 1 hectare controls. AFIT-01 was applied with a fertilizer spreader to treated plots at the rate of 10 kg/ha (containing 5 kg A2085 and 5 kg A2321). Control areas received not inoculated sterile sorghum applied in the same manner. The treated maize was at stem elongation, growth stage (BBCH 35-39), at treatment and AFIT-01 was applied to both the crop and soil during application. In each field, the treatment and control each were replicated three times with each approximately 0.34 ha. At commercial ripening (moisture content 22–24%), 10 ears from each replicate plot were randomly collected, de-husked, manually shelled, and the kernels dried (45 °C, 3 days) and milled to produce flour. This resulted in a total of 6 samples (3 replicates for treated plots and 3 for controls) of maize flour from each field.

#### 4.3.3. Recovery of Applied Atoxigenic Strains

*Aspergillus flavus* enumeration was carried out on the maize flour with dilution plate technique on Modified Rose-Bengal Agar (MRBA; [47]) (3.0 g sucrose, 3.0 g NaNO_3_, 0.75 g KH_2_PO_4_, 0.25 g K_2_HPO_4_, 0.5 g MgSO_4_·7H_2_O, 0.5 g KCl, 10.0 g NaCl, 1 mL of Adye and Matales micronutrients [48], 0.025 g Rose Bengal, 0.05 g chloramphenicol, 0.05 g streptomycin, 0.01 g dichloran (Sigma-Aldrich, St. Louis, MO, USA), 20.0 g Bacto agar (Difco Laboratories, Detroit, MI, USA), 1000 mL water). Briefly, ground maize (5 g) was suspended in 45 mL sterile 0.01% Tween 80 on a rotary shaker (300 rpm, 20 min). Aliquots (300 μL) of an appropriate dilution of the resulting suspension were spread on MRBA (*n* = 3) and incubated in the dark for 3 days at 31 °C. Members of *Aspergillus* section *Flavi* were identified by colony morphology. Colony-forming units (CFU) were recorded to facilitate quantification of *A*. *flavus* on the crop.

Ten discrete *A*. *flavus* colonies, from 2 independent isolations, were recovered from each sample, transferred on 5/2 agar, incubated for 5–7 days at 31 °C [35] and saved in sterile water vials [32]. Single spore colonies were used to estimate the relative abundance of the candidate biocontrol agents released in the maize fields based on frequencies of the agents’ vegetative compatibility groups as previously [23]. Compatibility between the isolates recovered and the two VCGs applied was evaluated using complementation tests with nitrate non-utilizing auxotrophs (nit^−^ mutants; [49]). Briefly, nit^−^ mutants formed on centrally inoculated Czapek-Dox Agar (CZ; 30.0 g sucrose, 3.0 g NaNO_3_, 0.5 g K_2_HPO_4_, 0.5 g KH_2_PO_4_, 0.5 g MgSO_4_·7H_2_O, 0.5 g KCl, 20.0 g Bacto agar, 1000 mL water) amended with potassium chlorate (25.0 g/L) and rose Bengal (0.05 g/L) at pH 7.0 [23]. Chlorate-resistant sectors were incubated on CZ amended with 15.0 g/L of potassium chlorate (Sigma-Aldrich, St. Louis, MO, USA) for 3 days at 31 °C to stabilize the mutants and then on 5/2 agar to produce sporulating cultures. Plugs of the sporulating cultures were stored in sterile distilled water in glass vials. Compatibility between the released VCGs (IT006 and IT019) and isolates recovered from maize flour was tested on starch medium (3.0 g NaNO_3_, 1.0 g K_2_HPO_4_, 0.5 g MgSO_4_·7H_2_O, 0.5 g KCl, 36.0 g dextrose (Sigma-Aldrich, St. Louis, MO, USA), 20.0 g starch (Difco Laboratories, Detroit, MI, USA), 20.0 g Bacto agar (Difco Laboratories, Detroit, MI, USA), pH 6.0, 1000 mL water; [50]) following the procedure previously described [32].

### 4.4. Aflatoxin Quantification

Maize flour samples from pin bar inoculation (3 replicates per sample) and natural contamination experiments (3 replicates per treatment, 2 treatments per field), resulting from the maize kernel milling (above), were homogenized and analyzed for mycotoxins. Aflatoxins were analyzed according to the method of Stroka et al. [51]. Briefly, 25 g of flour was extracted with 250 mL methanol:water (80:20, *v*/*v*) in a 250 mL Erlenmyer flask on with rotary-shaker (90 rpm, 45 min). The extract was passed through Whatman #4 (Whatman International Ltd., Maidstone, Kent, UK) filter paper and diluted 5 mL filtrate: 45 mL distilled water and passed through an immunoaffinity column (R-Biopharm Rhône Ltd, Glasgow, UK). Aflatoxins were eluted from the column with 2.5 mL methanol. The eluate, concentrated to 1 mL under a gentle stream of nitrogen, was brought to 2 mL with acetonitrile:water (25:75, *v*/*v*); the extract was then filtered (HV 0.45 μm, Millipore Corporation, Bedford, MA, USA) and analyzed by high performance liquid chromatography (HPLC). The HPLC instrument consisted of two PU-1580 chromatography pumps, a Jasco AS 1555 sampling system, a FP 1520 fluorescence (Jasco Corporation, Tokyo, Japan) detector with a post-column derivatization system. A Superspher RP-18 column (Merck, Darmstadt, Germany) was used at ambient temperature with a mobile phase of water:methanol:acetonitrile (64:23:13, *v*/*v*/*v*) at 1.0 mL/min. The detector was set at λ_ex_ = 365 nm and λ_em_ = 440 nm. The analyses had a limit of detection of 0.02 μg total aflatoxins/kg [52].

### 4.5. Fumonisin Quantification

Fumonisins B_1_ and B_2_ were analyzed according to the method of Visconti et al. [53] in samples collected in natural contamination experiments. Briefly, fumonisins were extracted from 10 g of maize flour with 50 mL of acetonitrile:methanol:water (25:25:50, *v*/*v*/*v*), in a 250 mL Erlenmyer flask on an orbital shaker for 45 min. After centrifugation, (4500× *g*, 6 min), the supernatant was recovered and the pellet was subjected to a second extraction and the centrifugation was repeated. Extracts were combined, filtered, and a 2 mL aliquot was diluted with 20 mL 0.1 M phosphate-buffered saline prior to being passed through an immunoaffinity column (R-Biopharm Rhône, Glasgow, UK). Fumonisins were eluted with 6 mL methanol and then concentrated to 2 mL under a gentle stream of nitrogen. Analyses were carried out using a liquid chromatography-mass spectrometry LC-MS/MS (Thermo-Fisher Scientific, San Jose, CA, USA) system. The limit of detection for the analyses was 10 μg total fumonisins/kg maize flour [52].

### 4.6. Verification of Fungal Identities

In order to verify identities of the atoxigenic *A*. *flavus* evaluated in the current study, these fungi were compared with the two atoxigenic *A*. *flavus* currently utilized as active ingredients in commercial biopesticides for the prevention of aflatoxin contamination. Mating-type (*MAT*) genes were characterized according to Ramirez-Prado et al. [54], and allele sizes at 17 SSR loci were characterized according to Grubisha and Cotty [55]. The active ingredients in the biopesticide products Aflaguard^®^ (NRRL21882; [56]) and *Aspergillus flavus* AF36 (NRRL18543; [22]) were included as standards for comparison. DNA was produced by the method of Callicott and Cotty [44] from a 7 day old culture at 31 °C on 5/2 agar. Briefly, spores from 7 day old cultures were rubbed from the 5/2 agar surface with a sterile cotton swab and transferred in 450 μL of lysis buffer (30 mM Tris buffer, 10 mM EDTA, 1% SDS, pH 8.0). Spore suspensions were incubated (60 °C, 800 rpm, 1 h) in a ThermoMixer (Eppendorf, Westbury, NY, USA) followed by centrifugation (14,000× *g*, 30 min). Supernatant (370 μL) was combined with an equal volume of 4 M NH_4_ Acetate, pH 4.8 and 740 μL of cold absolute ethanol, incubated at −20 °C for 30 min, and centrifuged (14,000× *g*, 5 min). After drying, the pellet was suspended in 25 μL sterile water, and DNA concentration was determined with a spectrophotometer (NanoDrop 1000, Thermo Scientific, Wilmington, DE, USA). Amplification of the *MAT1-1* gene was carried using the primers M1F (ATTGCCCATTTGGCCTTGAA) and M1R (TTGATGACCATGCCACCAGA) and the *MAT1-2* gene with the primers M2F (GCATTCATCCTTTATCGTCAGC) and M2R (GCTTCTTTTCGGATGGCTTGCG) [54].

PCR reactions were conducted in 20 μL using Accupower^®^ Hoststart (Bioneer, Alameda, CA, USA) PCR Pre Mix tubes with 5 ng genomic DNA, 0.25 μM each primer, and 1.5 mM MgCl_2_. PCR amplification was in a MyCycler thermocycler (Bio-Rad Laboratories, Richmond, CA, USA) with an initial 95 °C, 5 min step followed by 40 cycles of 95 °C, 30 s; 54 °C, 60 s; 72 °C, 45 s and final extension at 72 °C for 5 min. Amplicons were visualized with SYBR Gold after 1% agarose gel electrophoresis. *Aspergillus flavus* AF70 and NRRL21882 were used as positive controls for *MAT1-1* and *MAT1-2* genes, respectively [57,58].

Microsatellite loci amplifications followed the method of Grubisha and Cotty [55] utilizing loci AF8, AF11, AF13, AF16, AF17, AF18, AF22, AF28, AF31, AF33, AF34, AF42, AF43, AF53, AF54, AF55, AF63, AF64, and AF66. Fragment analyses of amplicons were performed with an ABI 3730 DNA Analyzer (Thermo Fisher Scientific, Foster City, CA, USA) at the Genetics Core Facility of the University of Arizona, Tucson, USA. The LIZ500 size standard (Thermo Fisher Scientific, Foster City, CA, USA) and G5 dye set were used. Allele sizes were called using GeneMarker version 1.7 (SoftGenetics, LLC, State College, PA, USA).

Mating type analysis and microsatellite characterization were repeated twice.

### 4.7. Statistical Analyses

All data was subjected to analysis of variance (ANOVA) using the software IMB SPSS Statistics 21 (IMB, Somers, NY, USA). Mean separations were performed with Tukey’s honest significant difference test (*p* = 0.05; [59]).

Percent reduction in aflatoxin B_1_ in the wound inoculation experiment was calculated as [1 − (aflatoxin in maize co-inoculated with both aflatoxin-producer and atoxigenic strains/aflatoxin in maize inoculated with the aflatoxin producer alone)] × 100; in the field experiment as [1 − (aflatoxin in maize treated with AFIT-01/aflatoxin in untreated maize)] × 100. Data on aflatoxin B_1_ and percent reduction were ln and arcsin transformed, respectively, prior to analyses. Fields with aflatoxin B_1_ concentrations below 1 μg/kg in both treatments were not considered for the analyses. *Aspergillus flavus* populations in the maize flour are reported as log (10) transformed colony forming units (CFU)/g dry maize flour.

## Figures and Tables

**Table 1:** Evaluation of Italian atoxigenic *Aspergillus flavus* strain A2085 and A2321 to reduce aflatoxin B_1_ contamination in a wound inoculation ears trials.

Inoculum ^a^	Year 2012			Year 2013		
	Aflatoxin B_1_ (μg/kg) ^f^	Transformed (ln + 1)	Reduction (%) ^h^	Aflatoxin B_1_ (μg/kg) ^f^	Transformed (ln + 1)	Reduction (%) ^h^
Unwounded ^b^	0.7	0.53		0.6A	0.45	
A2092 ^c^	1415.4	7.10		132.9	4.87	
A2085 ^d^	0.9	0.57		1.4	0.78	
A2321 ^d^	0.0	0.00		3.8	1.55	
A2092 + A2085 ^e^	96.2	4.57	93.2	2.3	1.05	98.3
A2092 + A2321 ^e^	1381.9	7.03	ns ^J^	176.7	4.99	ns
Not inoculated ^g^	ND ^i^	ND		1.2	0.67	

^a^ Ears were inoculated at BBCH69 using a device consisted of three needles, arranged in a triangle, dipped in a 10^5^ spore/mL spore suspension; ^b^ Ears naturally contaminated; ^c^ A2092 an aflatoxin-producing genotype of *A*. *flavus* originating from Italy serves here as the positive control; ^d^ A2085 and A2321 are atoxigenic genotypes of *A*. *flavus* originating from Italy; ^e^ Co-inoculation of aflatoxin-producing and atoxigenic strains; aflatoxin-producer A2092 was inoculated immediately before the atoxigenic strain; ^f^ Aflatoxin B_1_ concentration at harvest; ^g^ Ears wounded without inoculation; ^h^ Percentage of aflatoxin B_1_ reduction was calculated as [1 − (total aflatoxin in co-inoculation/total aflatoxin in A2092)] × 100; ^i^ ND = not determined; ^J^ ns = not significant.

**Table 2:** Quantity of *Aspergillus flavus* populations in maize kernels from eight fields after harvest and percent of the *A*. *flavus* community consisting of the two applied vegetative compatibility groups (VCGs) IT006 and IT019.

Location ^a^	District ^b^	CFU/g ^c^		VCGs (%) ^e^			
				Control		Treated	
		Control ^d^	Treated ^d^	IT006	IT019	IT006	IT019
Canaro (1)	Rovigo (RO1)	6.60	6.74	56.7	26.7	76.7	20.0
Dosolo	Mantova (MN1)	6.67	6.64	60.0	33.3	66.7	16.7
Moglia	Mantova (MN2)	4.82	5.95	53.3	16.7	60.0	30.0
Piano di Rivà	Rovigo (RO3)	5.09	5.52	73.3	16.7	40.0	46.7
Canaro (2)	Rovigo (RO2)	6.68	6.73	46.7	33.3	73.3	23.3
Fidenza	Parma (PR)	6.06	6.38	35.0	35.0	55.0	40.0
Legnago (1)	Verona (VR1)	6.82	7.18	60.0	17.7	56.7	33.3
Legnago (2)	Verona (VR2)	6.57	6.93	43.3	23.3	63.3	30.0
	Average	6.16	6.51	53.5	25.2	61.9	30.0

^a^ Site experiment localizations; ^b^ Political district; ^c^ Colony forming units (CFU) of *Aspergillus flavus* in ground corn after harvest expressed as log (10); ^d^ Untreated (control) and treated with 10 kg/hectare (ha) of *Aspergillus Flavus* ITaly 01 (AFIT-01) biopesticide; ^e^ Percentage of the *A. flavus* population infecting the maize belonging to vegetative compatibility group IT006 (VCG of A2085) and IT019 (VCG of A2321).

**Table 3:** Fumonisin B_1_ + B_2_ and aflatoxin B_1_ contamination of maize from eight fields treated with AFIT-01 biopesticide.

Location ^a^	District ^b^	Fumonisin B_1_ + B_2_ (mg/kg) ^c^		Aflatoxin B_1_ (μg/kg) ^c^		Aflatoxin B_1_ Reduction (%) ^d^
		Control	Treated	Control	Treated	
Canaro (1)	Rovigo (RO1)	0.1	0.1	<1.0	<1.0	-
Dosolo	Mantova (MN1)	5.0	5.0	<1.0	<1.0	-
Moglia	Mantova (MN2)	0.1	0.2	<1.0	<1.0	-
Piano di Rivà	Rovigo (RO3)	0.6	1.4	<1.0	<1.0	-
Canaro (2)	Rovigo (RO2)	0.1	0.1	37.9	0.2	94.8
Fidenza	Parma (PR)	7.9	11.1	8.6	1.5	83.7
Legnago (1)	Verona (VR1)	1.8	4.2	87.2	6.6	92.8
Legnago (2)	Verona (VR2)	2.1	2.2	150.7	8.4	94.5
Average		2.2	3.0	71.1	4.2	92.3

^a^ Site experiment localizations; ^b^ Political district; ^c^ Fumonisins and aflatoxin B_1_ concentrations at harvest in replicate plots not treated (control) and treated with 10 kg/ha of AFIT-01 biopesticide; ^d^ Percentage of aflatoxin B_1_ reduction was calculated as [1 − (aflatoxin B_1_ in maize treated with AFIT-01/aflatoxin B_1_ in maize untreated)] × 100.

**Table 4:** Size of amplicons for each of 16 microsatellite loci for the two atoxigenic genotypes of *Aspergillus flavus* used in the current tests and for two atoxigenic genotypes registered in the USA as active ingredients of biopesticides directed at preventing aflatoxin contamination.

Strain ^a^	Locus Name															
	AF8	AF11	AF13	AF16	AF17	AF22	AF28	AF31	AF42	AF43	AF53	AF54	AF55	AF63	AF64	AF66
A2085	166 ^b^	135	141	169	367	144	119	312	150	399	131	161	181	127	161	271
A2321	168	126	128	169	364	144	119	312	143	399	131	161	172	127	161	269
NRRL18543	177	162	161	191	353	188	119	309	162	385	134	169	174	135	211	269
NRRL21882	168	138	141	169	353	144	119	312	146	402	131	161	181	127	161	267

^a^ A2085 and A2321 are the active ingredients of AFIT-01; NRRL18543 and NRRL21882 are the active ingredients of AF36 and Aflaguard^®^ biopesticide, respectively; ^b^ Size expressed in base pairs.
