# Correction: Mauro, A., et al. Biological Control Products for Aflatoxin Prevention in Italy: Commercial Field Evaluation of Atoxigenic *Aspergillus flavus* Active Ingredients. *Toxins* 2018, *10*, 30

**DOI:** 10.3390/toxins11020117

**Published:** 2019-02-14

**Authors:** Antonio Mauro, Esther Garcia-Cela, Amedeo Pietri, Peter J. Cotty, Paola Battilani

**Affiliations:** 1 International Institute of Tropical Agriculture, Dar es Salaam P.O. Box 34441, Tanzania; a.mauro@cgiar.org; 2 Applied Mycology Group, Environment and AgriFood Theme, Cranfield University, Cranfield, Bedford MK43 0AL, UK; m.e.garcia-cela@cranfield.ac.uk; 3 Institute of Food Science and Nutrition, Università Cattolica del Sacro Cuore, 29100 Piacenza, Italy; amedeo.pietri@unicatt.it; 4 United States Department of Agriculture, Agricultural Research Service, School of Plant Sciences, University of Arizona, Tucson, AZ 85721, USA; Peter.Cotty@ARS.USDA.GOV; 5 Department Sustainable Crop Production, Università Cattolica del Sacro Cuore, 29100 Piacenza, Italy

The authors wish to make the following correction to their paper [1]. 

In Section 2.3, Mating-Type and Microsatellites, the first sentence should be replaced with “Amplification of the mating type genes revealed that strain A2321 has the idiomorph MAT1-2 and strain A2085 has the idiomorph MAT1-1.”

We apologize for any inconvenience caused to readers of Toxins by this change.
